# Incorporating preference uncertainty in best worst scaling

**DOI:** 10.1371/journal.pone.0315705

**Published:** 2025-01-30

**Authors:** Francisco J. Areal, Rubén Perez

**Affiliations:** 1 Newcastle Business School, Northumbria University, Newcastle upon Tyne, United Kingdom; 2 Centre for Rural Economy, School of Natural and Environmental Sciences, Newcastle University, Newcastle upon Tyne, United Kingdom; 3 Centro Universitario Ciudad de Mendoza, Universidad Nacional de Cuyo, Mendoza, Argentina; University of South Florida, UNITED STATES OF AMERICA

## Abstract

In this paper, we enhance the Best-Worst Scaling (BWS) method by incorporating participants’ preference uncertainty into the conventional BWS, known as case 1. In this context, respondents are tasked with making trade-offs among a set of items of interest. Applying this novel extended BWS method to a sample of Argentinian wine consumers (n = 342), we aim to a) provide a more informative elicitation of consumers’ relative preferences for 16 wine attributes; b) identify the level of uncertainty with each of the attributes, exploring differences between the most and least important wine attributes influencing purchasing wine; and c) compare the results of the extended BWS with the standard BWS. Our findings indicate variability in uncertainty levels on the importance of wine attributes when purchasing wine within and across attributes. Moreover, accounting for participants’ preference uncertainty can alter the ranking of preferences obtained through the standard approach. This alteration is due to both accounting for preference uncertainty itself as well as the uncertainty indicator used. Although this approach is a way to mitigate biases associated with respondents’ preference certainty, it is recommended that preference uncertainty heterogeneity is investigated using different indicators.

## Introduction

Best-Worst Scaling (BWS) is a survey-based method used to study and measure individual choice (i.e. measure individual preferences of multiple choices) [1]. In BWS, survey respondents are presented with sets of items and asked to identify the “best” (most preferred or most important) and the “worst” (least preferred or least important) within each set. This process is repeated over several choice cards, providing information from which researchers can derive the individual preference for each of the items presented. The relevance of BWS lies in the fact that it forces respondents to make trade-offs between the items being evaluated, and reduces biases associated with the use of traditional rating scales such as Likert scales, which measures people’s attitudes, opinions, or perceptions by asking respondents to rate their level of agreement or disagreement on a multi-point scale (e.g. 5 point scale, 7 point scale), and ultimately provides a measure of the relative strength of item preferences [1, 2].

The use of the BWS method has gained popularity for evaluating an individual’s relative preferences regarding attributes or items, involving the selection of the most and the least attractive characteristics from a set of choices [1, 3]. The BWS method is important and holds appeal due to its capacity to capture trade-offs and preferences within complex decision-making scenarios, outperforming Likert scale ratings [4]. This approach mitigates issues such as response bias and inadequate differentiation among items [5]. Notably, the BWS method’s simplicity in allowing respondents to choose the best and worst options among alternatives has contributed to its widespread application in diverse research domains, overcoming response biases that may be present in approaches such as rating scales [6]. Examples include health care [2, 7], transportation [8], consumer’s ethical beliefs [9], environmental studies [10–12], forest management [13], energy [14], urban planning [15], education [5, 16], public policy [17], and in the analysis of perceptions of risks [18].

Despite the merits of employing the BWS method in capturing an individual’s relative preferences it is important to note that it does not inherently account for preference uncertainty among BWS respondents. This crucial aspect remains unaddressed within the standard framework of the BWS method. Respondents may have incomplete knowledge about their true preferences for product attributes (e.g. attributes for which they may be less familiar such as environmental and social aspects associated with products) and provide uncertain answers to eliciting methods (e.g. best-worst scaling). Consumer uncertainty may stem from a lack of information (i.e. respondents may feel they need more information about the attributes [19]); choice overload (i.e. respondents are presented with too many choices may hamper their intrinsic motivation and confuse them [20, 21]); psychological factors such as fatigue and cognitive biases (e.g. confirmation bias) [22]. Importantly, the use of heuristics, which are used by consumers to overcome lack of information and expertise on a product, can lead to biases when uncertainty is present under decision making [23]. All these aspects can contribute to respondents facing challenges in decision-making, potentially compromising the reliability of elicited preferences. One effective approach to account for preference uncertainty involves assessing the level of knowledge among respondents. In preference analysis, respondents’ knowledge can be divided into subjective and objective knowledge categories. Subjective knowledge pertains to respondents self-assessment of their understanding about the topic of study, typically measured in Likert scales gauging respondents’ belief levels. Objective knowledge involves an external evaluation through the testing of respondents’ knowledge via a set of questions related to the study’s subject matter. Conversely, although in applied preference analysis, respondents’ subjective and objective knowledge is typically not taken into account [24], with some exceptions [25, 26], preference uncertainty has been addressed by incorporating follow up questions that inquire about respondents’ certainty regarding their stated preferences (e.g. using a scale, such as a 10-point scale) [24, 27–29]. Neglecting preference uncertainty can result in an overestimation of respondents’ willingness to pay, particularly in non-market valuation methods such as contingent valuation and choice experiments [30, 31]. Therefore, understanding and addressing consumer uncertainty in the methods used to assess an individual’s preferences is crucial for obtaining robust preference elicitation.

This paper introduces and implements a method designed to address preference uncertainty within the context of BWS responses. We employ this extended BWS approach in a study involving Argentinian wine consumers, gathering data on their attribute preferences and associated preference uncertainty using a questionnaire. This paper contributes by 1) devising an extended BWS method to enhance understanding of trade-offs and respondent preferences in complex decision-making scenarios; and 2) evaluating the influence of preference uncertainty in attribute/option selection on respondents’ preferences, comparing results obtained from standard BWS with the extended BWS in a sample of Argentinian wine consumers.

The remainder of the paper is structured as follows. We begin by introducing the extended BWS method, detailing the data collection process, and outlining the experimental design. The following section presents and discusses the results, with the last section concluding.

## Data and methods

### The extended BWS

A variety of approaches, methods and techniques have been used to study consumer wine preferences encompassing sensory analysis; statistical examination of survey data, often through questionnaires and choice experiments; as well as qualitative methods like focus groups, and in-depth interviews. The literature extensively deploys hedonic price models to elucidate the impact of various wine attributes, such as wine variety and quality, grape type, and region of origin. Consequently, the associations between wine consumers’ characteristics and their preferences have been explored through different lenses, including the emotion-based perspective [32, 33]; consumers’ personality traits [34]; wine attitudinal and behavioural differences of consumers differences [35]; frequency of use [36]; and demographic differences [37]. In addition, research has also been conducted on consumers’ perceptions of wine attributes such as production origin, and the sustainability of wine production [38, 39].

To assess the Argentinian consumer preferences for a set of Argentinian wine characteristics, we employed and compared two approaches. We used a) a simple standard BWS approach, known as case 1, where respondents are required to make trade-offs between a set of items of interest [1, 3]; and b) a novel extended BWS, which was developed to account for respondent’s preference uncertainty. A consumer’s relative preferences are revealed through their response choices. The theoretical framework which underlies the analysis of BWS data is Random Utility Theory (RUM), which is widely used in the economic and econometric literatures. RUM assumes that consumers select between alternatives that maximise their utility [1, 40].

Louviere and Flynn [1] show that summarising standard best and worst data can be done by obtaining a simple scale that subtracts the worst count for each attribute (i.e. the sum of every time each attribute has been selected as worst) from the best count for that attribute (i.e. the sum of every time each attribute has been selected as best).

$$BWS\;score_{a}=\sum_{i=1}^{N}B_{ia}-\sum_{i=1}^{N}W_{ia}$$ (1)

where *a* refers to an attribute, with *a = 1*, *…*, *16*; *B*_*ia*_ takes a value of 1 if attribute *a* is selected as “the most important” by respondent *i* and 0 otherwise; *W*_*ia*_ takes a value of 1 if attribute *a* is selected as “the least important” by respondent *i* and 0 otherwise.

We account for respondents’ uncertainty in their choices by asking them “On a scale 0–100 how sure are you that you have selected the best attribute?” and “On a scale 0–100 how sure are you that you have selected the worst attribute?” after each of the choice cards are presented to them and they have made their selections.

$$BWS\;score_{a}=\sum_{i=1}^{N}\alpha B_{ia}-\sum_{i=1}^{N}\omega W_{ia}$$ (2)

where *α* represents the level of certainty that respondent *i* has in their selection of “the most important” attribute from each choice card; and *ω* represents the level of certainty that respondent *i* has in their selection of “the least important” attribute from each choice card. The values of *α* and *ω* range between 0 and 1 and are obtained by dividing the value given by respondents on the 0–100 scale by 100.

In order to account for uncertainty, we use a respondent’s self-reported uncertainty about the choices made in each of the choice cards presented to them.

### Control vs. treatment group

We split the sample into one control group (n = 185) and one treatment group (n = 158), enabling us to examine whether making respondents aware of their uncertainty in answering questions had any effect on their BWS attribute scores and ranking. For this, we compared the standard BWS attribute scores between the treatment and control groups. The treatment group was presented with 16 choice cards, with 6 attributes in each. After choices were made for each choice card, participants were asked to self-report how sure they were of the choices made (i.e. most and least important attribute). The follow-up questions that respondents in the treatment group were asked after each choice card was shown and selections were made were “On a scale 0–100 how sure are you that you have selected the **best** attribute?” and “On a scale 0–100 how sure are you that you have selected the **worst** attribute?”. The control group was presented with 16 choice cards with 6 attributes in each, according to Tables 3, 4, with no follow-up questions made on how certain they were on the choices made in the choice cards presented.

We calculated and compared the BWS attribute scores (i.e. the preference (un)certainty scores) and rankings for the standard BWS and extended BWS that accounts for (un)certainty for each of the 16 attributes considered. We focus on the preference (un)certainty median scores for the most and least important attributes in consumers’ decision-making, since preference (un)certainty distributions are not normally distributed, with the median being less influenced by extreme values, thus providing a more robust measure of central tendency. To compare standard and extended BWS scores, we use the sign test for the median This test is a conservative non-parametric test that imposes minimal assumptions on the data distribution. Although we focus on the preference (un)certainty median scores, we also present the results for the preference (un)certainty mean scores for the most and least important attributes in consumers’ decision-making for completeness, and test whether the preference (un)certainty distributions differ using the non-parametric Wilcoxon rank sum test (also known as the Mann-Whitney U test).

The treatment group is divided into two subgroups: a) participants who are relatively more certain about their wine attribute preferences; and b) participants who are relatively less certain, to examine any differences which may exist in the BWS scores and rankings between these subgroups and the control group. It is expected that respondents belonging to the less certain group may state different preferences than the relatively more certain group since their relative uncertainty about choices may cause them to be indecisive. Rankings may be altered compared to the more certain participants, given this indecisiveness. Finding such differences would suggest that heterogeneity in respondents’ level of certainty about their choices matters in attribute preference elicitation. There are two ways in which we measure the relative certainty level of group participants: a) calculating the certainty median of the individual’s average certainty across wine attributes, dividing the sample into those above and below the median of their average certainty across the attributes; and b) calculating the individual’s coefficient of variation:

$$CV_{i}=\frac{\;Standard\;deviation\;of\;the\;certainty\;level\;across\;the\;attributes_{i}}{\;Mean\;of\;the\;certainty\;level\;across\;the\;attributes_{i}}\times 100.$$

### Questionnaire and data collection

The questionnaire included an introduction to the questionnaire where all participants were presented with the BWS task as follows–“Imagine you are in your favourite wine shop to buy a bottle of wine to drink at home with family and/or friends. You will then be shown a series of tables with 6 attributes or characteristics of the wine in each table. Please select the characteristic that would influence you the most and the characteristic that would influence you the least when considering the purchase of a bottle of wine in the shop. Some attributes or characteristics shown may not be common but please consider all the characteristics as if they were possible”. The questionnaire also included the BWS experiments (control and treatment groups with follow-up questions on preference uncertainty) and socio-demographic characteristics (gender, income, age, and education level).

Snowball sampling, a popular non-probability convenience sampling technique [41], was conducted via an electronic questionnaire using Qualtrics during January and February, 2023. We previously conducted a small survey to investigate wine consumer preferences in March 2022 and piloted the questionnaire in December 2022. Participants were invited to participate through social media platforms, where initial participants recruit others, creating a “snowball” effect. Leveraging social networks allows for amplifying the reach of our survey to respondents, providing access to individuals who would not easily be identifiable through random sampling [42], as well as accessing geographically dispersed populations [43]. We advertised on WhatsApp and Twitter to ensure the inclusion of diverse perspectives [44]. A brief explanation of the research was provided to participants with a link to the questionnaire. A total of 343 valid responses were used in the analysis. We recorded 740 responses but 397 were incomplete (i.e. respondents started but did not finish the questionnaire).

Generalisability of our results is limited given the selection bias inherent in the initial snowball sample that relied upon our personal network [45]. Nonetheless, it is noteworthy that the resulting sample includes all age groups, both genders (Table 1), and various geographical locations (Table 2). We acknowledge that the sample may underrepresent the lower income group and overrepresent the highest income group.

**Table 1:** Sample socio demographics.

	Total	%
***Gender***		
Female	105	0.31
Male	237	0.69
***Income (pesos)***		
<120,000	26	0.08
120,001–180,000	67	0.20
181,000–240,000	62	0.18
241,000–300,000	52	0.15
300,001+	136	0.40
***Age***		
18–30	41	0.12
31–50	211	0.62
51+	91	0.27
***Education level***		
Primary	1	0.00
Secondary	49	0.14
Higher education (university studies)	293	0.85

**Table 2:** Sample and census population geographical distribution.

Region	Sample	Census
Gran Buenos Aires	47.23%	30.44%
Pampeana	27.11%	34.53%
Cuyo	23.32%	7.43%
North West	1.46%	12.77%
Patagonia	0.29%	5.62%
North East	0.58%	9.21%

The sample includes a comprehensive representation from diverse regions, as illustrated in Table 2 which compares our sample with the most relevant available 2022 census data. It is noteworthy that we have gathered information from 14 provinces. Within the Gran Buenos Aires area, the sample encompasses 19 municipalities.

### Experimental design

The experimental design consisted of 16 choice cards presenting 16 different options for each respondent (i.e. respondents were shown 16 sets of 6 attributes). Respondents are asked to select their most and least preferred wine attribute within each set that they consider. The selection of the 16 options was based on literature review and a small consumer survey conducted to explore individual preferences (Table 3). Note that attributes 7 and 12 are not true attributes, but respondents’ attributes to the particular wine. Regarding the literature on consumers’ preferences for wine attributes Stanco, Lerro [46] and Casini, Corsi [47] studied the wine attribute preferences of Italian consumers. Casini and Corsi [47] found that direct, personal, and sensorial experiences are the most important attributes when choosing wine. Stanco and Lerro [46] found that traditional attributes such as “geographical indications”, “grape variety”, “sustainable certification”, “vintage”, and “price”, are more important for consumers than novel attributes such as “canned wine”, “alcohol-free wine”, and “vegan wine”. Nunes and Madureira [48] found that “tested the wine previously”, “recommended by friends/relatives” and “region of origin” were the preferred attributes for the sample of Portuguese wine buyers studied.

**Table 3:** Attributes used in the best worst scaling.

Attribute number	Attribute	Reference
1	The grape variety	[47–53]
2	The price	[48–50]
3	The Argentine region of origin	[47–49, 51–53]
4	The ageing	[50]
5	The type of closure (e.g. cork, plastic or screw cap)	[50, 53]
6	The wine has been awarded a quality medal/award	[47, 48, 50, 52]
7	Have tested the wine before	[47–49, 51–53]
8	The wine has a certificate of environmental sustainability (e.g. organic/ecological)	[46]
9	The wine is on promotion in the shop/supermarket	[47–49, 51–53]
10	The alcohol content	[47, 49, 51–53]
11	The label provides information on how grapes are harvested (mechanically or by hand)	-
12	The wine has been recommended to you	[47–49, 51–53]
13	The brand name	[47–49, 51–53]
14	The label is attractive	[47, 48, 50–53]
15	The information on the back label	[47, 48, 51–54]
16	The wine has a certificate of rural sustainability (sustain economic and social activities in rural communities)	-

We used a Balanced Incomplete Block Design (BIBD) to construct a number of choice cards presented to Argentinian wine consumer respondents to assess their preferences for the set of 16 Argentinian wine characteristics or attributes.

A BIBD is a type of experimental design used when it is not practical to test all possible combinations of conditions. It assigns each attribute to various choice cards. This type of experimental design ensures that all attributes are compared in a balanced way (i.e. each attribute appears the same number of times across all choice cards), with not all attributes being present in each of them. Hence, BIBD is a) balanced since each attribute is paired with every other attribute an equal number of times across all choice cards; b) incomplete because not all attributes appear in every choice card; and c) block design since each choice card is a “block” that includes a subset of attributes.

To conduct BIBD the attributes need to be firstly identified and assigned into choice cards (blocks) so that each attribute appears the same number of times across all choice cards and every pair of attributes appears together in the same choice card an equal number of times.

Let *J* be the list of 16 wine attributes, and denote the individual attributes on the list by *j* = 1, 2, …, *J*. Let denote *k* and *b* as the fixed size of a choice card (i.e. number of attributes in a choice card) and the number of choice cards (blocks), respectively. Each attribute is shown *r* times and co-occurs with each other attribute λ times. To assign attributes to choice cards attributes can be numbered from 1 to J. BIBDs generate *b* choice cards from which respondents make their evaluations (i.e. select their most preferred and least preferred attribute). BIBDs are available in a variety of sources [1, 55], but also can be generated using multiple statistical packages (e.g. bwsTools, and AlgDesign for R) [56–58]. Here we used the AlgDesign package for R [56] to generate a BIBD (*J* = 16, *b* = 16, *r* = 6, *k* = 6, λ = 2)-design. This means that our BIBD consisted of 16 choice cards with 6 attributes in each choice card, where each attribute appears equally often (6 times); and they concur twice with other attributes (Table 4). We selected a BIBD with 16 choice cards and 6 options because, with 16 attributes, this design was the one with the least number of choice cards possible (easing the respondents’ burden of going through too many choice cards) whilst dealing with a reasonable number of choices within each choice card (6).

**Table 4:** Balanced incomplete block designs (BIBDs)– 16 choices & 6 options.

Choice card	Attribute number					
1	5	6	7	10	14	15
2	4	9	10	12	14	16
3	1	8	9	11	14	15
4	2	3	9	10	13	15
5	1	2	5	7	9	16
6	1	7	8	10	12	13
7	2	6	8	13	14	16
8	5	6	9	11	12	13
9	1	3	6	10	11	16
10	1	2	4	6	12	15
11	2	4	5	8	10	11
12	3	4	6	7	8	9
13	3	5	8	12	15	16
14	4	7	11	13	15	16
15	2	3	7	11	12	14
16	1	3	4	5	13	14

**Fig 1:**
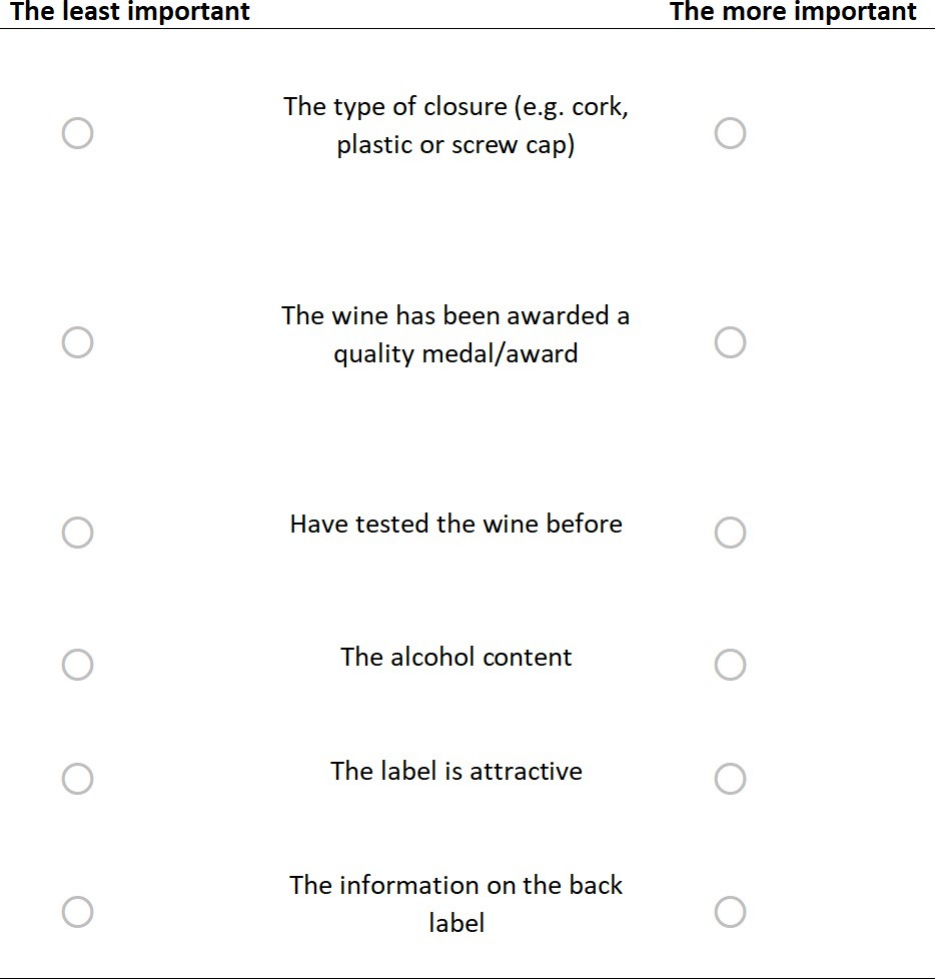
Choice card containing 6 wine attributes.

The case 1 of the best-worst scaling method presents respondents with a set of 16 choice cards, each showing 6 wine attributes, and asked them to select the best and worst attributes based on their preferences. Fig 1 shows one of the choice cards used in this study.

### Inclusivity in global research

The Newcastle University Ethics Committee granted the ethical approval for this research. Participants were informed that the aim of the research was to learn about Argentinean consumer’s preferences in Argentinean wine consumption. They were encouraged to express their views freely, were assured that their views would be valued, and that there were no right or wrong answers to the questions asked. They were informed that we did not collect any names or personal details. Participants were asked to voluntarily participate in the survey, and were made aware that they could withdraw from the survey at any point. They were informed that by completing the survey, they acknowledge they understand the terms and conditions of participating in the study, and that they accepted these terms. Additional information regarding the ethical, cultural, and scientific considerations specific to inclusivity in global research is included in the Supporting Information (S1 Checklist).

## Results and discussion

This section presents three type of results including those associated with the level of certainty respondents have on their selection of the most and least important wine attributes when purchasing a bottle of wine; results showing the most and least important wine attributes for the sample of Argentinian wine consumers and the score and ranking comparisons between the control and treatment groups, their relative level of (un)certainty, as well as between the use of the standard and extended BWS approach; and the results sensitivity to uncertainty indicators. Finally the section points out the limitations of the study.

Our results indicate that the most important wine attributes are grape variety, previous recommendations, prior testing, price, and the Argentinian region of origin. We also found that individuals are generally certain about what attributes are least and most important when purchasing wine. However, they tend to be more certain about what attributes are least important than those that are most important. We also found that when integrating uncertainty into the BWS analysis, both BWS scores and rankings vary in comparison to the standard BWS analysis. When incorporating uncertainty, scores and rankings also vary depending on the method used to measure uncertainty.

### Level of certainty respondents have on their selection the of most and least important wine attributes

**Fig 2:**
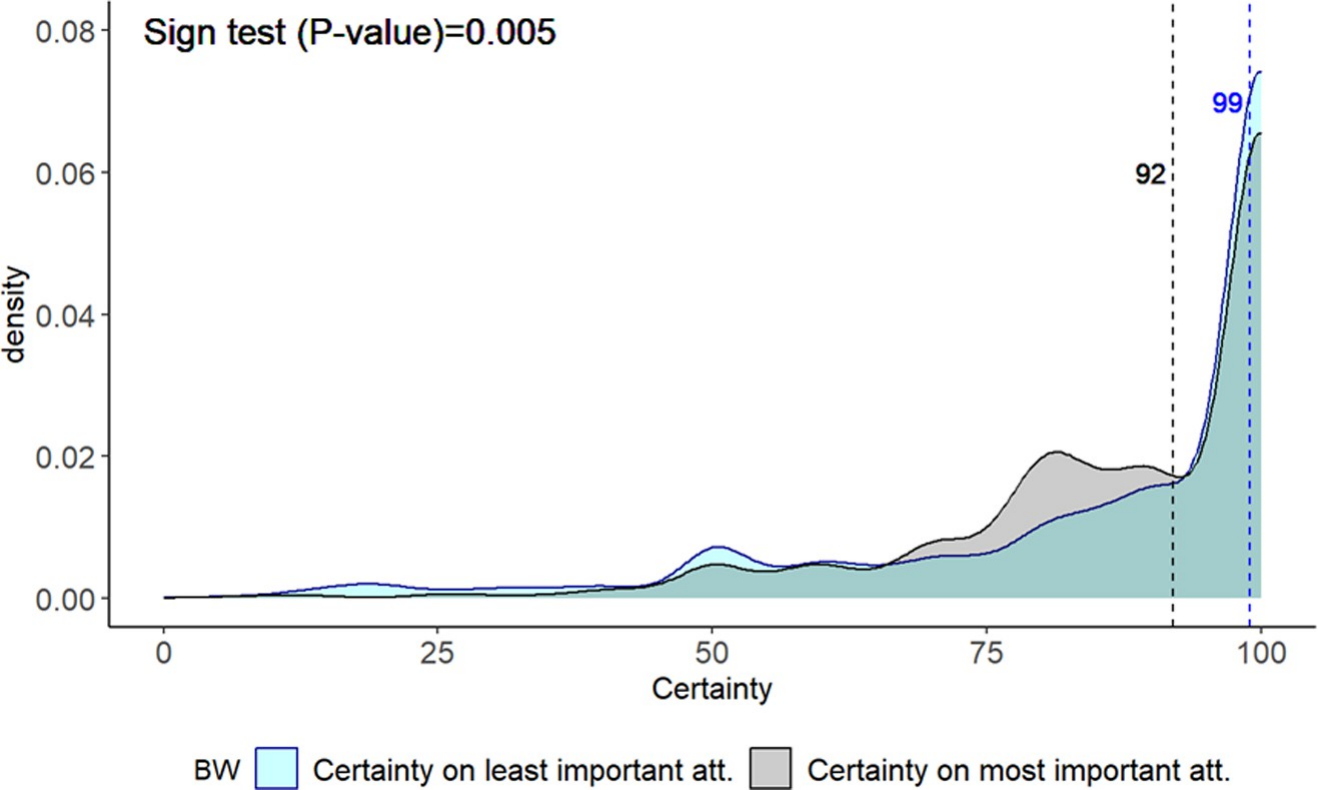
Density plot for the level of certainty when selected as most and least important (all attributes).

Overall, respondents were relatively certain about the choices made. The certainty median scores for the least and most preferred attributes were 99 and 92, respectively (i.e. 50% of respondents have 99% and 92% levels of certainty on what their least and more preferred attributes are, respectively). However, many respondents were uncertain or very uncertain about their choices. Thus, 9.4% and 5.1% of respondents were 50% or less certain about the least and most important attribute chosen, respectively. A two-sided sign test indicates a statistical difference between the median of the certainty score of attributes selected as least important and the median of the certainty score of attributes selected as most important (p<0.01). We found that out of the 2,528 pairs of observations (158 participants in the treatment group were shown 16 cards), there were 777 pairs (30.7%) where the level of certainty about the least important attribute was greater than the level of certainty about the most important attribute, 661 (26.2%) where the level of certainty about the least important attribute is less than the level of certainty about the most important attribute, and 1,090 (43.0%) where the level of certainty about the least important attribute is the same than the level of certainty about the most important attribute. In Fig 2, the density plots illustrate the level of certainty associated with all attributes identified as both most important and least important in the decision-making process regarding the purchase of a bottle of wine.

**Fig 3:**
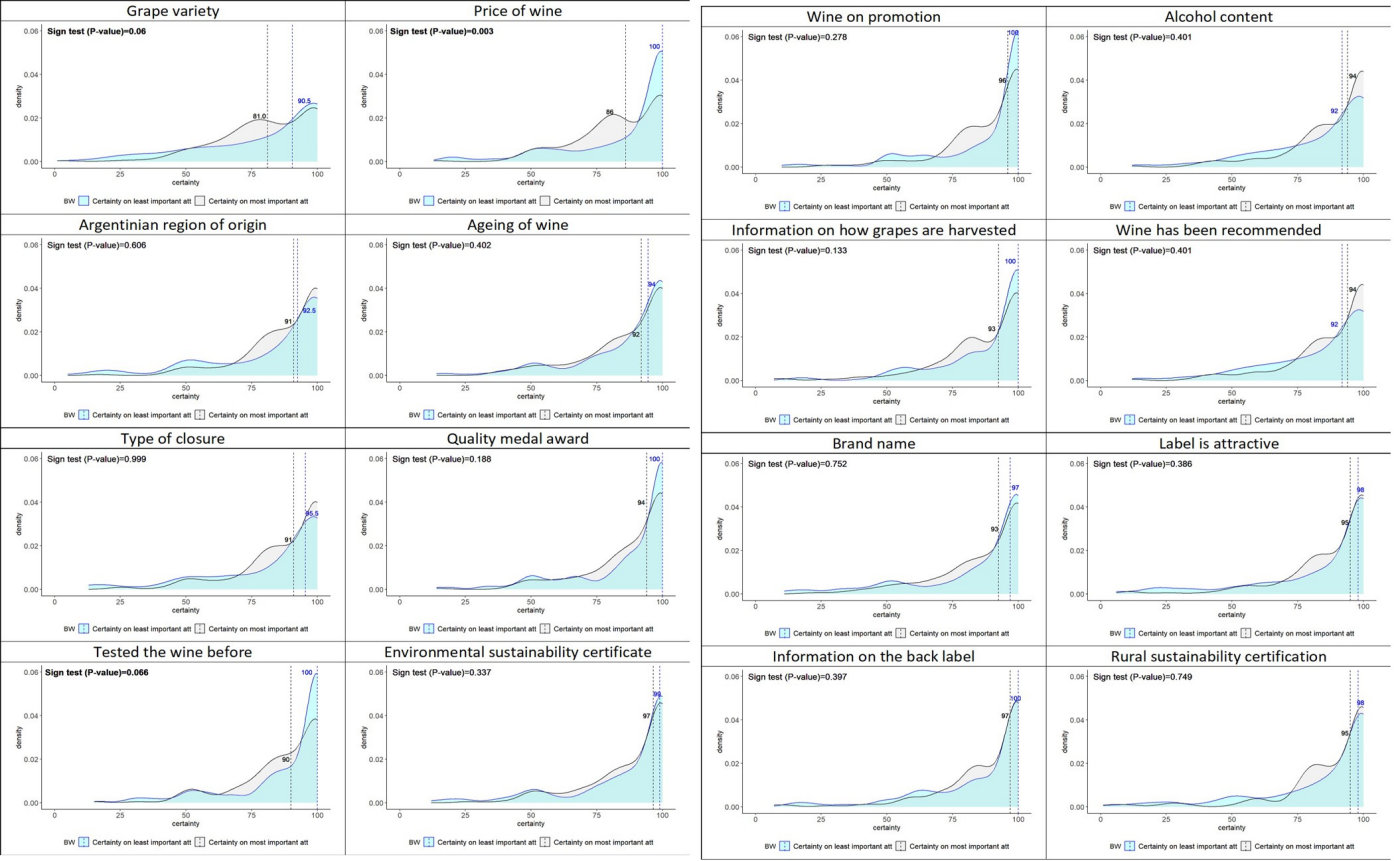
Density plots and medians for the level of certainty per attribute when selected as most and least important.

Fig 3 shows the density plots of the level of certainty associated with each of the attributes identified as both most important and least important in the decision-making process regarding the purchase of a bottle of wine. Additionally, Fig 3 shows the two-sided sign test. The findings reveal variation in the median level of certainty among respondents regarding the attribute selected as both most and least important across the sample. While a majority of participants exhibit a relatively high certainty level in their choices, a significant portion still expresses notable uncertainty, as evidenced by the extended left tail of the distributions.

Participants exhibit a tendency to assign a higher level of certainty when identifying the least important attributes compared to the most important ones. However, it is noteworthy that the scores for most important attributes surpass those assigned to least important attributes. Thus, the average certainty score for most and least important attributes are 87.2 and 85.8, respectively. More specifically, the top five attributes that respondents were least certain about when identifying them as the most important attribute are: grape variety, wine price, whether they had tasted the wine before, the Argentinian region of origin, and the type of closure. The top 5 attributes that respondents were least certain about when selected as the least important attribute are grape variety, wine has been recommended, alcohol content, Argentinian region of origin and ageing of wine.

Table 5 shows that BWS scores and ranking per attribute obtained using the standard approach for the control and treatment groups as well as the extended approach for the treatment group. Our findings indicate two effects: a) when integrating uncertainty (treatment group) into the analysis, specifically by including self-reported uncertainty information through additional follow-up questions for each BWS card in the extended BWS approach, both scores and rankings may vary in comparison to the control group, where uncertainty information is not acquired; and b) scores and rankings may also exhibit variations due to the BWS method employed, the extended BWS approach or the standard BWS. The first effect may be due to treatment group respondents becoming more aware of their level of certainty and thinking more carefully about their choices. However, for this particular sample, such variations are relatively small. Alterations in ranking are dependent on the proximity of the standard BWS scores and the level of certainty respondents have on the choices they make.

**Table 5:** Scores and ranking comparison between standard and certainty (extended) BWS methods.

Attribute number	Attribute	Standard BWS control	Standard BWS treatment	Certainty BWS treatment	Standard BWS control ranking	Standard BWS ranking	Certainty BWS ranking
1	The grape variety	531	469	422	1	1	1
12	The wine has been recommended to you	430	398	351	2	2	2
7	Have tested the wine before	373	344	305	3	3	3
2	The price	207	211	175	4	4	4
3	The Argentine region of origin	181	102	95	5	5	5
13	The brand name	56	38	35	6	6	6
6	The wine has been awarded a quality medal/award	5	30	31	7	7	7
4	The ageing	-26	21	22	8	8	8
15	The information on the back label	-28	-51	-36	9	10	10
9	The wine is on promotion in the shop/supermarket	-57	-13	-20	10	9	9
11	The label provides information on how grapes are harvested (mechanically or by hand)	-185	-190	-163	11	11	11
8	The wine has a certificate of environmental sustainability (e.g. organic/ecological)	-231	-249	-216	12	14	13
16	The wine has a certificate of rural sustainability (sustain economic and social activities in rural communities)	-245	-254	-222	13	15	15
14	The label is attractive	-255	-246	-220	14	13	14
5	The type of closure (e.g. cork, plastic or screw cap)	-296	-203	-173	15	12	12
10	The alcohol content	-460	-400	-351	16	16	16

### Most and least important wine attributes when purchasing wine

We ranked wine attributes from most to least important using the best worst scaling scores through both the standard BWS and the extended BWS (taking into account the level of (un)certainty).

In the context of Argentinian consumer preferences for wine purchasing decisions, the top five most crucial attributes, ranked in descending order, include grape variety, previous recommendations, prior testing, price, and the Argentinian region of origin. Notably, four out of these five attributes are associated with a relatively lower respondent certainty. Previous results on grape variety are mixed with some research indicating it as an important attribute (53) whereas other research found it relatively less important (42, 49). Having tasted the wine previously is usually a top attribute for wine consumers across different countries (43, 51). The region of origin has also been found to be important for French Spanish, Italian and Australian wine consumers (42, 43, 49, 51, 53). Recommendations by friends and relatives were also found as important attributes in consumers wine selection for Spanish and Australian consumers (49, 53), whereas price was found to be important for Spanish consumers (49). The 5 least important attributes in wine purchasing decision-making are the alcohol content, type of closure, attractiveness of the label, the wine having a certificate of rural sustainability, and the wine having a certificate of environmental sustainability. Alcohol content was found to be the least important attribute in wine choice across different countries (50, 51). The extrinsic wine attributes such as the type of closure, labelling, knowing about how the grapes have been harvested, rural and environmental sustainability certification, being on promotion, information on the back label, quality awards and the brand name are all found to be relatively less important attributes when selecting wine for respondents. Being on promotion, back label, quality awards and brand name were also found to be relatively less important for Italian, Australian and Israeli consumers (42, 53).

### Sensitivity to uncertainty indicators

Collecting information on the respondents’ level of certainty allowed us to group respondents according to their relative level of certainty (e.g. relatively more certain vs. relatively less certain) on their most preferred attribute.

Tables 6 and 7 show differences in score and ranking between relatively more and less certain respondents on their most preferred attribute using a) the certainty median of the individual’s average certainty across attributes and b) the coefficient of variation as indicators of respondents’ level of certainty, respectively. When using the median of individual’s average certainty across attributes to group respondents, we found that the grape variety, the wine being recommended and having tested the wine before come as the most important attributes for both groups, whereas the alcohol content comes as the least important attribute. When using the median of the individual certainty’s coefficient of variation to group respondents into relatively more and less certain the grape variety, the wine being recommended and having tested the wine before also come as the top 3 attributes, although there are slight differences between less and more certain groups when using the standard BWS compared to the extended BWS. Alcohol content also came as the least important attribute when using the individual certainty’s coefficient of variation to group respondents into relatively more and less certain. Differences in scores and rankings were found between groups and indicators used of respondents’ level of certainty. For instance, wine promotion in the shop/supermarket is an attribute that is relatively important for those who are more certain about their responses (rank 5^th^) as opposed to those who are less certain on their responses (rank 10^th^) when using the certainty median as an indicator. Also, the wine been awarded a quality medal/award was relatively more important for more certain respondents (rank 6^th^) than for less certain respondents (10^th^). However, when comparing results shown in Tables 6 and 7 differences in ranking and score are found to be sensitive to the method used to distinguish between relatively more and less certain on their attributes preferences. For example, the Argentine region of origin is a relatively more important wine attribute for respondents who are more certain about their preferences than for those who are less certain about their preferences, when using the individual’s average certainty across attributes to group respondents into relatively more and less certain about their relative preferences. However, when using the median of the certainty’s coefficient of variation to group respondents into more and less certain, Argentine region of origin is relatively more important for respondents who are less certain than for those who are more certain about their preferences. This means that the use of different uncertainty indicators may lead to different preference rankings, which can have important implications for decision making (e.g. marketing strategies). Different uncertainty indicators capture/represent different ways to interpret preference uncertainty. Hence, it is important to use a range of indicators to present these different interpretations of preference uncertainty. Decision makers then have a broader understanding of individuals’ preferences and their uncertainty about them, which can help to take informed decisions. It is worth noting that it is possible that the differences in most preferred attributes between less and more certain respondents may be due to factors that determine their expressed level of confidence. For instance, maybe people who are confident in their answers are more frequent consumers and maybe for such consumers, the relevant attributes are different than for those than are less frequent wine consumers.

**Table 6:** Scores and ranking comparison between relatively more and less certain respondents on the most preferred attribute–Median of the certainty’s average across attributes.

Attribute number	Attribute	Standard less certain group BWS score	Extended less certain group BWS score	Standard more certain group BWS score	Extended More certain group BWS score	Standard less certain group BWS ranking	Extended less certain group BWS ranking	Standard more certain group ranking	Extended more certain group BWS ranking
1	The grape variety	229	188	240	234	1	1	1	1
12	The wine has been recommended to you	183	142	215	209	2	2	2	2
7	Have tested the wine before	168	135	176	169	3	3	3	3
2	The price	138	103	73	72	4	4	5	5
9	The wine is on promotion in the shop/supermarket	39	28	-52	-49	5	5	10	10
3	The Argentine region of origin	28	22	74	73	6	6	4	4
13	The brand name	8	3	30	29	7	7	7	7
4	The ageing	2	3	19	19	8	8	8	8
15	The information on the back label	-9	-5	-42	-35	9	9	9	9
6	The wine has been awarded a quality medal/award	-14	-10	44	40	10	10	6	6
11	The label provides information on how grapes are harvested (mechanically or by hand)	-97	-78	-93	-88	11	11	11	12
5	The type of closure (e.g. cork, plastic or screw cap)	-107	-82	-96	-87	12	12	12	11
14	The label is attractive	-109	-87	-137	-132	13	13	15	15
16	The wine has a certificate of rural sustainability (sustain economic and social activities in rural communities)	-121	-98	-133	-127	14	14	14	14
8	The wine has a certificate of environmental sustainability (e.g. organic/ecological)	-134	-107	-115	-109	15	15	13	13
10	The alcohol content	-202	-157	-198	-188	16	16	16	16

**Table 7:** Scores and ranking comparison between relatively more and less certain respondents on the most preferred attribute–Median of the certainty’s coefficient of variation.

Attribute number	Attribute	Standard less certain group BWS score	Extended less certain group BWS score	Standard more certain group BWS score	Extended More certain group BWS score	Standard less certain group BWS ranking	Extended less certain group BWS ranking	Standard more certain group ranking	Extended more certain group BWS ranking
1	The grape variety	239	225	230	198	1	1	1	1
7	Have tested the wine before	176	163	168	142	2	3	3	3
12	The wine has been recommended to you	175	164	223	187	3	2	2	2
3	The Argentine region of origin	80	76	22	19	4	4	5	6
2	The price	75	68	136	106	5	5	4	4
4	The ageing	27	27	-6	-7	6	6	9	10
6	The wine has been awarded a quality medal/award	24	23	6	7	7	7	8	9
13	The brand name	22	21	16	13	8	8	6	7
9	The wine is on promotion in the shop/supermarket	-25	-27	12	7	9	10	7	8
15	The information on the back label	-26	-25	-25	-16	10	9	10	11
11	The label provides information on how grapes are harvested (mechanically or by hand)	-80	-74	-110	-93	11	11	13	13
5	The type of closure (e.g. cork, plastic or screw cap)	-95	-88	-108	95	12	12	12	5
8	The wine has a certificate of environmental sustainability (e.g. organic/ecological)	-134	-125	-115	-96	13	13	14	14
16	The wine has a certificate of rural sustainability (sustain economic and social activities in rural communities)	-136	-128	-118	-97	14	14	15	15
14	The label is attractive	-150	-137	-96	-85	15	15	11	12
10	The alcohol content	-168	-156	-232	-203	16	16	16	16

### Limitations

Despite the valuable insights provided in this study, several limitations must be acknowledged. First, the non-random sample used may not be fully representative of the population of Argentinian wine consumers, which could limit the generalisation of the findings. Hence, cautions should be exercised when extrapolating our findings in other contexts. While the sample may not be fully representative of the broader population, the findings remain relevant and provide valuable insights specific to the group studied. These results offer useful information that can inform future research. Secondly, the attributes were presented to the respondents in the order that are listed in Table 4, without randomisation of the order, which can create some biases if the order in which they are presented impacts the respondents’ answers. This study shows one way to account for an individual’s preference uncertainty in their reported preferences. In expression (2), we assumed that individuals can measure their level of uncertainty in their choices precisely. However, individuals could also be uncertain about their stated level of uncertainty, which could impact the results obtained.

## Conclusions

A novel approach to account for preference uncertainty using BWS is presented and applied to a sample of Argentinian wine consumers. The approach extends the standard BWS method by including a respondent’s preference (un)certainty on attributes. This approach is shown to yield different scores and rankings of attributes compared to the standard approach when accounting and not accounting for preference (un)certainty through the use of self-reported uncertainty follow up questions.

Although most respondents may be certain about their responses, there may always be respondents who are uncertain about their preferences and, in particular, regarding some individual attributes. Not accounting for an individual’s preference uncertainty may lead to biases and inaccurate representations of true preferences and poor forecasting of decision-making. The approach presented here offers a way to account for preference uncertainty and mitigate the issues it causes. We advocate for the use of this approach to evaluate the impact of preference uncertainty in decision-making since it may make respondents more focused on the task.

We found that attribute preference uncertainty may vary across attributes. The extension proposed here allows for the accounting of (un)certainty by adding follow-up questions for each of the choice cards presented to respondents. Accounting for attribute preference uncertainty may alter the BWS ranking of preferences and, therefore, the recommendations made. This new method offers researchers a way to mitigate biases associated with respondents’ preference (un)certainty. In addition, it is recommended that preference uncertainty heterogeneity is investigated using different indicators.

We use self-reported (un)certainty, asking participates to indicate their level of uncertainty on the choices made on a 0–100 scale. Other alternative (un)certainty indicators could be used. For instance, response times, where longer response times may suggest respondents needed more time to think, indicating uncertainty, other scales (e.g. Likert scales) that then will need to be transformed into weighting parameters *α*, *ω* bounded between 0 and 1 for the most (least) preferred respectively. The transformation could be conducted using normalisation methods (e.g. max-min scaling). Although we applied this method to the simplest BWS case (case 1), similar approaches can be developed for case 2 and case 3 as well as other choice experiments to mitigate bias associated with preference uncertainty.

The findings in this study also have policy implications. The use of the extended BWS proposed here can have relevant policy implications in areas such as public health, environmental policy and transportation, for example, by providing more accurate representations of an individual’s true preferences and more nuanced, adaptive policy interventions. The approach and findings presented here indicate that rigid, one-size-fits-all policies may not be appropriate in many situations. With the use of the method presented here, policy makers can use the knowledge of individuals’ preference uncertainty to design more flexible, adaptive, and inclusive policies that better reflect the complexity around individual preferences. The use of information provided by this approach by policy makers could enhance policy effectiveness and public acceptance.

## Supporting information

S1 Checklist (DOCX)
